# Evidence for a delay in diagnosis of Wilms’ tumour in the UK compared with Germany: implications for primary care for children

**DOI:** 10.1136/archdischild-2015-309212

**Published:** 2016-03-06

**Authors:** Kathy Pritchard-Jones, Norbert Graf, Harm van Tinteren, Alan Craft

**Affiliations:** 1 University College London Institute of Child Health, London, UK; 2 Department of Paediatric Oncology/Hematology, Saarland University Hospital Homburg, Homburg, Germany; 3 Head Biometrics Department, Netherlands Cancer Institute—Antoni van Leeuwenhoek Hospital, Amsterdam, Netherlands; 4 Department of Child Health, Royal Victoria Infirmary, Newcastle upon Tyne, UK

**Keywords:** Routes to diagnosis, Outcomes research, Oncology

## Abstract

The UK has a longstanding system of general practice which provides the vast majority of primary care, including that for children. It acts as a 'gatekeeper' to more specialist care. Parents may also use accident and emergency departments as their first point of medical contact for their children. Outcomes in the UK for many conditions in children appear to be worse than in comparable European countries where there is direct access to care by paediatricians. We have therefore looked at pathways to diagnosis and compared outcomes in the childhood kidney cancer, Wilms' tumour, which has been treated in the UK and Germany within the same clinical trial for over a decade. We find that Wilms' tumours are significantly larger in volume and have a more advanced tumour stage at diagnosis in the UK compared to Germany. There is a small (∼3%) difference in event free and overall survival between the two countries. Our data suggest that the system of primary care for children in the UK is less likely to result in the incidental finding of an abdominal mass in a child with no or vague symptoms. This may be a reason for the poorer outcome.

## Introduction

There has been much interest in recent years in comparative cancer survival data between different European countries for adults and children. Concerns have been raised about the possibility of worse outcomes for children with Wilms’ tumour in the UK since the publication of the ACCIS (Automated Childhood Cancer Incidence and Survival) report in 2006.^1^ This project, coordinated by the International Agency for Research in Cancer, Lyon, brought together all of the available population-based childhood cancer registry data in Europe for a systematic analysis of incidence and survival by tumour type. For children with Wilms’ tumour diagnosed in the period 1988–1997, there was a small but significant difference in 5-year overall survival (OS) between the British Isles (83%, 95% CIs 78% to 85%) compared with countries in the North and West of Europe that is, Scandinavia, Benelux, France and Germany (89%, 95% CIs 88% to 91%).

Survival differences may be due to one or a combination of factors including patient delay in presentation to medical attention, delay in recognition in primary care and referral for suspected cancer or variable application of ‘best practice’ diagnostic workup and treatments in secondary care.^2^

Wilms’ tumour in children is an ideal cancer in which to look at the influence of these factors in childhood cancer. It is one of the more common solid tumours, affecting 1 in 10 000 children. Treatment is standardised across Europe through the majority of cases being treated within the context of an international phase III clinical trial with full patient demographic, treatment and outcome data collected in a comparable way. This overcomes the limitation of population-based registry studies where only OS can be compared with ease.^3^ Since March 2002, the UK and Republic of Ireland participated as a national group (Children's Cancer and Leukaemia Group, CCLG) in the International Society of Paediatric Oncology (SIOP) Wilms’ tumour (WT) 2001 trial, in which the majority of participating European countries do so at a near-population level.^4^

### National comparisons of patient outcomes within an international Wilms’ tumour clinical trial

The more detailed data in clinical trial databases allows comparisons of relapse-free survival as well as OS and the several factors that determine the overall burden of therapy each child requires. These in turn dictate the risk of long-term morbidity of their cancer treatment—a factor that is extremely important in this childhood cancer with an almost 90% cure rate and with survivors expected to live a near normal adult life expectancy. CCLG contributed 18% of the patients registered in the trial, that now includes >5000 patients with renal tumour in its database. Hence, for the first time in the 2000s, it has been possible to compare directly the patient characteristics, tumour stage distribution and outcomes of children diagnosed in the UK with a contemporaneous, virtually population-based trial recruitment in other Western European countries, all treated according to the same protocol. We have chosen to use Germany for the comparison with UK patients entered into the SIOP WT 2001 trial and study, as both national groups have additional data on the route to diagnosis of their patients, which is necessary to inform interpretation of any differences.

There is a marked and statistically significant difference in the distribution of tumour stage at diagnosis but not of histological risk group between patients registered in the UK compared with Germany (table 1). These two parameters are the main determinants of the risk-stratified treatment that is assigned to each patient in the trial. The adverse (higher) stage distribution in the UK is largely seen among localised tumours, the small excess of metastatic disease at presentation does not reach statistical significance. Tumour volume at diagnosis is also markedly increased in the UK compared with Germany (table 1).

**Table 1:** Characteristics of registered patients with Wilms’ tumour treated with preoperative chemotherapy according to the SIOP WT 2001 trial protocol in Germany and the UK and Republic of Ireland

Characteristic	GPOH		CCLG		p Value	Statistical test
Total number patients*	951		616			
Median age at diagnosis (months)	40.4		40.4		0.902	Rank-sum test
**Tumour stage distribution**		%		%	<0.001	χ^2^ (3 df)=73.64
Stage I	524	55.1	219	35.6		
Stage II	172	18.1	136	22.1		
Stage III	107	11.3	150	24.4		
Stage IV	148	15.6	111	18		
Stage I–III (all localised tumours)	803	84.4	505	82	0.227	χ^2^ (1 df)=1.46
Stage IV (metastatic tumours)	148	15.6	111	18		
**Histological risk group**						
Low risk	49	5.2	39	6.3	0.568	χ^2^ (2 df)=1.13
Intermediate risk	780	82.0	503	81.7		
High risk	122	12.8	74	12.0		
**Tumour size (mL)**						
Tumour volume at diagnosis	381.8		572		<0.001	Rank-sum test
IQR	197.8, 627.6		334.7, 903.7			

*The UK registered 18% of all patients until December 2011, when it closed the study to national recruitment, after the closure of the randomised trial arm, for which only a subgroup of all WT patients were eligible. All other European countries continue to register patients.

CCLG, Children's Cancer and Leukaemia Group; GPOH, Gesellschaft für Pädiatrische Onkologie und Hämatologie).

From this within-trial comparison, it can be seen that children with Wilms’ tumour in the UK are diagnosed with significantly more advanced disease than in a comparable western European country. There is no difference in the distribution of histological risk groups, suggesting that it is unlikely that there are significant differences in tumour biology within these populations. This increased burden of disease at diagnosis translates into a small difference in event-free survival (EFS) and OS between the German (5-year EFS 87.6% (95% CI 85.4% to 89.9%); 5-year OS 94.5% (95% CI's 92.9% to 96.2%) and UK (5-year EFS 84.5% (95% CI's 81.6% to 87.5%); 5-year OS 91.3% (95% CI's 89.0% to 93.7%) patients (see online supplementary figure S1). This survival difference of ∼3% appears confined to the children who present with non-metastatic disease. This would be sufficient to explain the small population-based survival differences seen between Germany and Great Britain in the ACCIS studies in the past.^1^

### Routes to diagnosis of Wilms’ tumour

One possibility for the less good outcome is that children in the UK are presenting later than they would do in other countries. In order to address this question, a retrospective case note audit was undertaken to examine the presenting symptoms and diagnostic interval of all patients presenting with WT to three major UK paediatric oncology centres, the Royal Marsden Hospital (RMH), Great Ormond Street Hospital (GOSH) in London and the Royal Victoria Infirmary, Newcastle (RVI). The analysis included all patients registered in one of three consecutive trials (UKW2, UKW3, SIOP WT 2001) that ran between 1986 and 2011.^5^ Patients were categorised into three groups and the time interval from first symptom to presentation to medical attention was recorded:

- *Diagnosed through screening* (either for a recognised Wilms’ tumour predisposition syndrome or investigation of multiple congenital abnormality, or detected during a routine child health check)
- *Presented with non-tumour related symptoms* (ie, an abdominal mass was detected as part of an astute clinician, usually a GP, fully examining a child whose parents were concerned about other symptoms, or a junior doctor detected the mass during clerking for a routine medical procedure)
- *Presented with tumour related symptoms*, most commonly a palpable abdominal mass, abdominal pain or haematuria

We have combined groups A and B into ‘asymptomatic’ for symptoms related to Wilms’ tumour, and group C is ‘symptomatic’. Eighty-five per cent of children were diagnosed in the symptomatic group and there was no difference between the three centres (GOSH 83%, RMH 87%, RVI 89%). Children in the symptomatic group were significantly older (median 3.3 years, IQR, 1.9, 4.7 years, range 0.1–16.6 years) than the asymptomatic group (median 2.2 years, IQR 1.0, 3.3; range 0.3–5.8 years; Wilcoxon rank-sum test, p<0.001). The time interval between the onset of symptoms and presentation to medical attention that led to onward referral for specialist oncological management was very short in all groups and was usually 1–2 days, median of 1 week (IQR, 0.3, 2.0 weeks; range 0–32 weeks) in the symptomatic group, showing that delay at this stage is not a major factor. There was a higher proportion of early stage tumours (72.7% stages I and II) in the asymptomatic group compared with those who were symptomatic (52.9%) stages I and II, (Fischer's exact test p=0.015).

This suggests that patients whose tumours are diagnosed following tumour related symptoms are more likely to have more advanced disease that requires a higher burden of therapy for stage III (local spread within the abdomen) and stage IV (distant spread or metastases). This includes risk-stratified use of doxorubicin, a chemotherapy agent carrying a risk of long-term cardiotoxicity, and radiotherapy, neither of which are required for stages I and II tumours, where the cancer is completely resected.

### Survival according to route to diagnosis

None of the 14 patients found by screening (group A) relapsed or died. Of the 39 found incidentally (group B), 6 (15%) relapsed and 3 died and of the 307 diagnosed with tumour related symptoms (group C), 53 (17%) relapsed and 33 (11%) died. There were no statistically significant differences in either EFS or OS between groups, even when groups A and B are combined and compared with group C. However, this result is likely to be due to the small number of events, as the significant difference in tumour stage between groups would be expected to translate into survival differences if larger numbers of patients were studied.

Within the participating national groups in the SIOP renal tumour study group trials, only Germany routinely collects data on route to diagnosis. Among 947 patients registered in the SIOP 93-01 trial (1994–2001), they found a higher proportion (27%) of children diagnosed either through screening (10%) or following assessment of non-tumour related symptoms (17%) (equivalent to our asymptomatic group) compared with only 14.7% in the three-centre UK study. There was a higher proportion of localised tumours detected in this category. Conversely, 18.9% of children presenting with tumour related symptoms had metastatic disease at diagnosis, similar to the proportion in the UK. The 5-year EFS was better for those in their equivalent of our ‘asymptomatic’ group (86%) than in the group with tumour related symptoms (82%), although the difference did not quite reach statistical significance (p=0.050) (see online supplementary figure S2).

### Possible reasons for differences observed

Similar international benchmarking comparisons of OS in adult cancer have suggested that poorer outcomes are seen in countries where general practitioners act as the gatekeeper to specialist care.^6^ Much emphasis has been placed on determining whether these survival differences are due to late presentation to medical attention or delayed diagnosis and referral after first presentation. Our retrospective audit data show that once a GP has a suspicion of an abdominal mass, they refer promptly and the system provides rapid access to diagnostic investigation and treatment. Our international benchmarking data within the same clinical trial provide good evidence that a higher proportion of children with renal tumours in Germany are diagnosed asymptomatically compared with the UK. This is associated with a lower tumour stage and better outcomes. We do not think that the problem is one of late referral after a suspicion of a tumour has been recognised, rather it reflects a failure to pick up relevant symptoms and signs during other healthcare contacts.

### Differences in national child healthcare practices

Our data suggest that the root of the poorer outcome could lie in the nature of child health practices in the UK. Fewer Wilms’ tumours are picked up incidentally perhaps because there is less routine examination of children or full physical examination of children with minor symptoms. The healthcare systems for children in the UK and Germany are radically different with the primary contact in Germany being a primary care paediatrician and in the UK a general practitioner.

In the UK developmental screening and routine health checks were radically reviewed by Hall and colleagues in 2000. They evaluated the effectiveness and pick-up rate of abnormalities at the multiple examinations which had previously been undertaken. Their conclusion in Health for All 4^7^ was that a routine physical examination should be undertaken for every newborn baby and again at around 6–12 weeks. They could find no justification to recommend any further routine examination before the child enters school at age 5 years and suggested that an examination then too is of questionable value. Most of the routine examinations are undertaken by health visitors who are public health nurses. A child's primary medical care is provided by a general practitioner. Hall suggested a move from the active ‘child health surveillance’ to one of a more passive ‘child health promotion’. Bellman and Vijeratnam asked whether we have in fact thrown the baby out with the bath water.^8^

In the UK less than 50% of general practitioners (GPs) who undertake primary care for children have received any formal paediatric training beyond that which they get in general practice training placements. Although many GPs have a great deal of experience of working with children, as they may constitute up to 40% of their routine workload, parents often choose instead to take their child to the Accident and Emergency department of a hospital.^9^ In Germany the vast majority of primary healthcare for children is provided by primary care paediatricians. They are office based and often have ultrasound scanning machines in their offices and are trained to use them. The routine health screening mandated and reimbursed is undertaken by these trained primary care paediatricians at seven time points in the first 2 years of life, then twice more up to primary school age.

### Conclusions and next steps

We suggest that the poorer outcome for children with Wilms’ tumour in the UK when compared with Germany may be due to a delay in detection resulting in children with larger and more advanced stage tumours which require a greater burden of therapy to treat successfully. While it would be difficult to justify a radical change in the way that children in the UK receive their primary care on the basis of Wilms’ tumour diagnosis alone, there are concerns that children in the UK have poorer outcomes for many illnesses and conditions.^10^ However, in these other disease areas it is more difficult to measure whether the differences lie in the standard of primary care for children. It is therefore essential that the UK continues to participate fully in the clinical studies and trials of the European clinical trial and study groups, where enhanced data on the route to diagnosis and burden of disease at diagnosis can be captured in a standardised fashion for international benchmarking and analysis of time trends. To continue to monitor this in a prospective fashion at a population level, we initiated in 2012, a clinical observational study, IMPORT (Improving Population Outcomes for Renal Tumours of childhood), that also investigates biomarkers for improved risk stratification. The registration form includes questions on each patient's route to diagnosis and time intervals, as suggested in the report of the Children and Young People's Health Outcome Forum.^11^ The median tumour volume at diagnosis for the first 218 patients registered in the IMPORT study (September 2012 to May 2015) remains elevated at 630 mL. This reinforces our earlier findings that one of the reasons for the poorer outcome seen for UK patients in the international comparative studies is likely to be later diagnosis in the UK.

We believe that our data could be used to justify a fresh look at the training requirements of general practitioners and health visitors in the way they look after children. Furthermore, consideration should be given to more integrated working with paediatricians in the community setting, as suggested in the recent call for a UK Child Survival Countdown initiative to tackle UK child survival in a European context.^12^ In 1976 the Court Committee report, *Fit for the Future*, recommended the strengthening of primary care for children by the training of general practitioner paediatricians but this did not find favour. We now present evidence that we should be reconsidering the way children are cared for in the National Health Service.

## Supplementary Material

Web legends

Web figure 1a

Web figure 1b

Web figure 1c

Web figure 1d

Web figure 1e

Web figure 1f

Web figure 2
