# Supplementary material for: Evidence for a delay in diagnosis of Wilms’ tumour in the UK compared with Germany: implications for primary care for children
Source: Arch Dis Child. 2016 Mar 6;101(5):417–20. doi: 10.1136/archdischild-2015-309212 (PMC4862069; doi:10.1136/archdischild-2015-309212)
Supplement: Web legends [file archdischild-2015-309212-s1.pdf]

Supplementary Figure legends

Fig 1: Event free (A, C, E) and Overall (B, D, F) survival curves for children with Wilms' tumour from the UK (CCLG) and Germany (GPOH) registered in the SIOP WT 2001 trial & study. All survival curves are for children with unilateral Wilms' tumour treated with pre-operative chemotherapy as per protocol. Panels A & B (all Wilms' tumours); C & D (localised Wilms tumours); E & F (metastatic Wilms tumours).

Fig 2: Event free survival by mode of diagnosis in the SIOP 93-01/GPOH* Wilms tumour trial and study. Cases detected by screening or at a routine child health check (Group A) or presenting with non-tumour related symptoms (Group B) are combined into one group and compared with the tumour-related symptoms Group C. *GPOH: German Paediatric Haemato-Oncology national group.
