# Supplementary figures and images for: Evidence for a delay in diagnosis of Wilms’ tumour in the UK compared with Germany: implications for primary care for children
Source: Arch Dis Child. 2016 Mar 6;101(5):417–20. doi: 10.1136/archdischild-2015-309212 (PMC4862069; doi:10.1136/archdischild-2015-309212)

# EFS by country

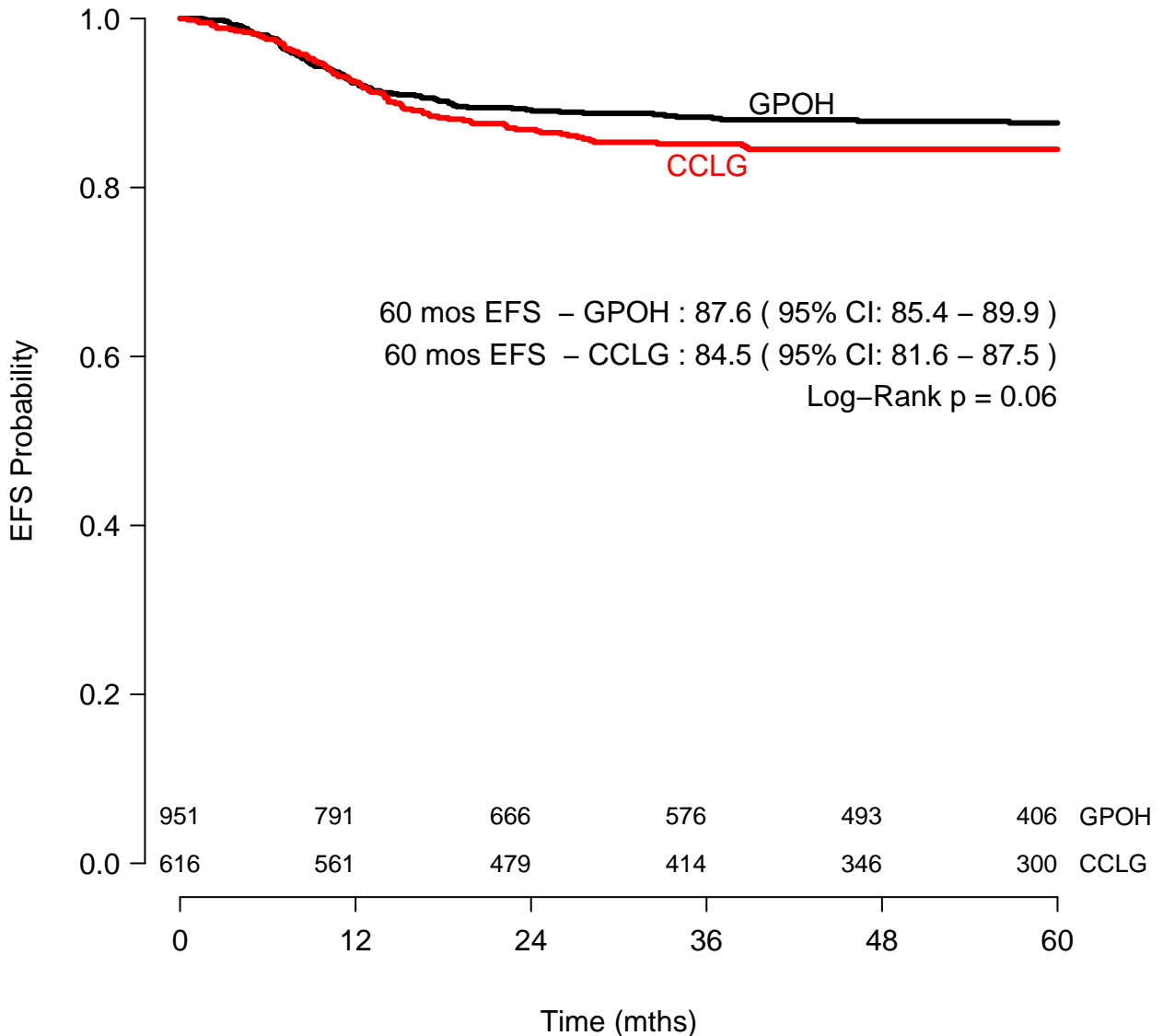

Supplement: Web figure 1a [file archdischild-2015-309212-s2.pdf]

## OS by country

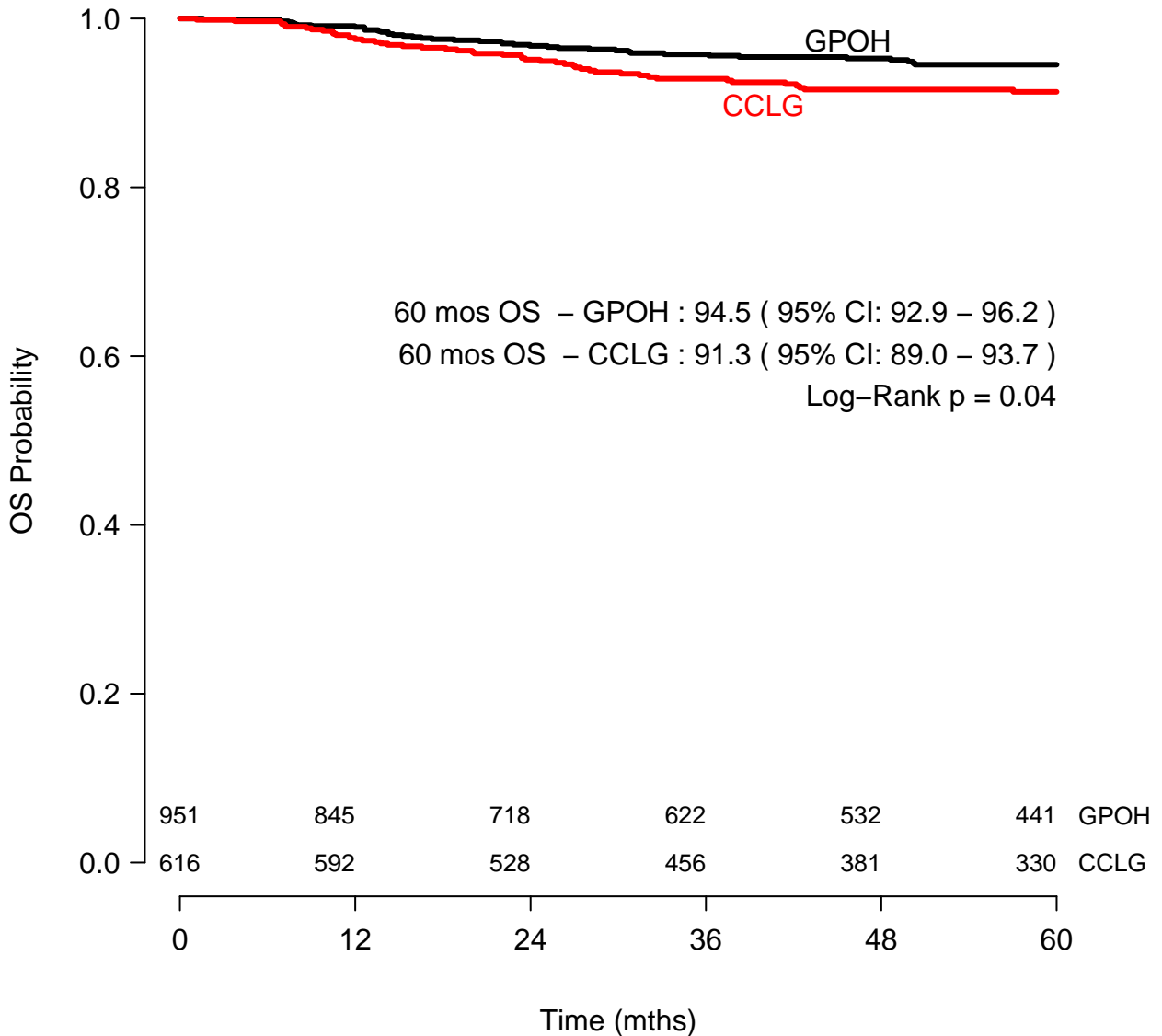

Supplement: Web figure 1b [file archdischild-2015-309212-s3.pdf]

## localised disease

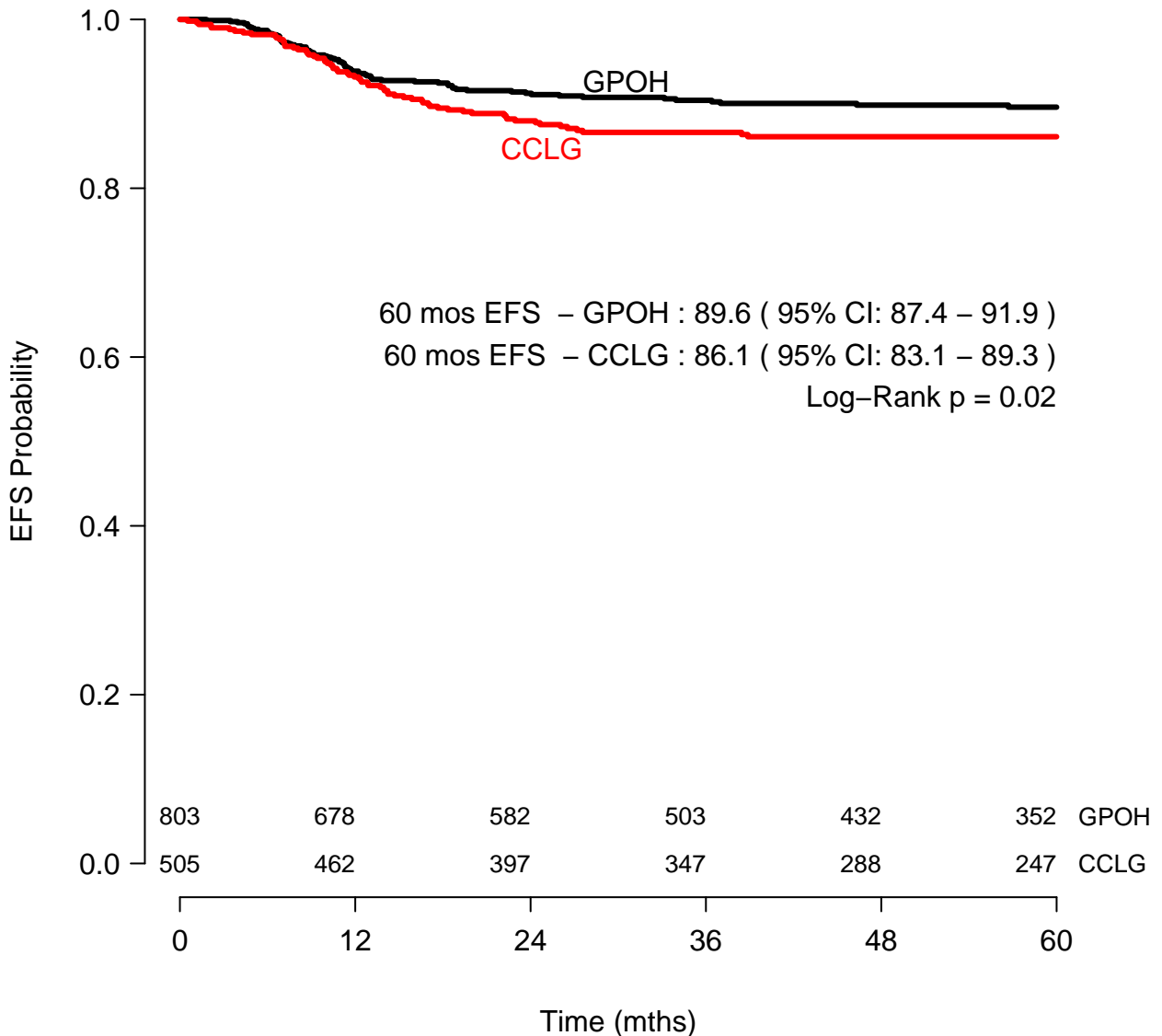

Supplement: Web figure 1c [file archdischild-2015-309212-s4.pdf]

## localised disease

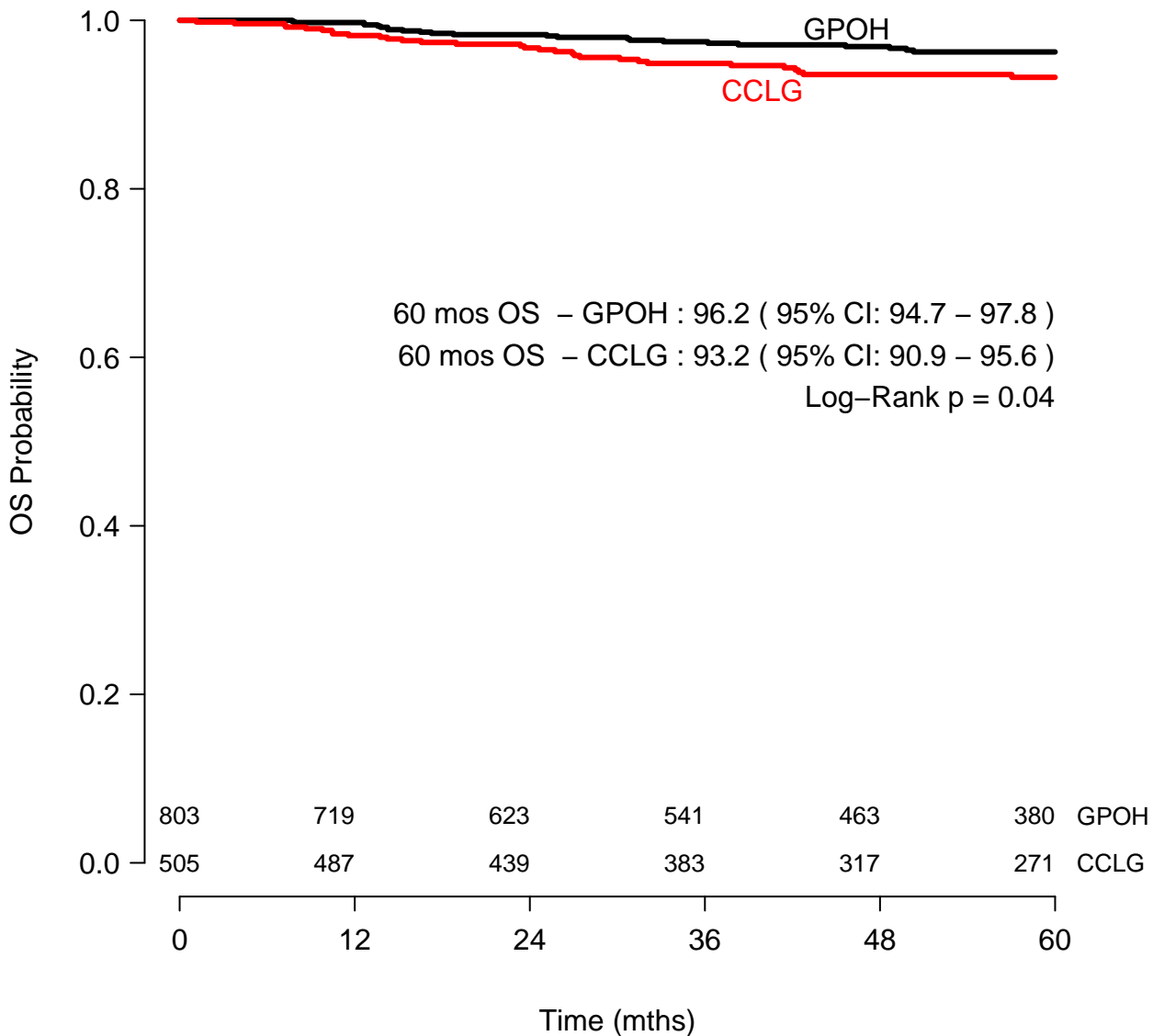

Supplement: Web figure 1d [file archdischild-2015-309212-s5.pdf]

## metastatic disease

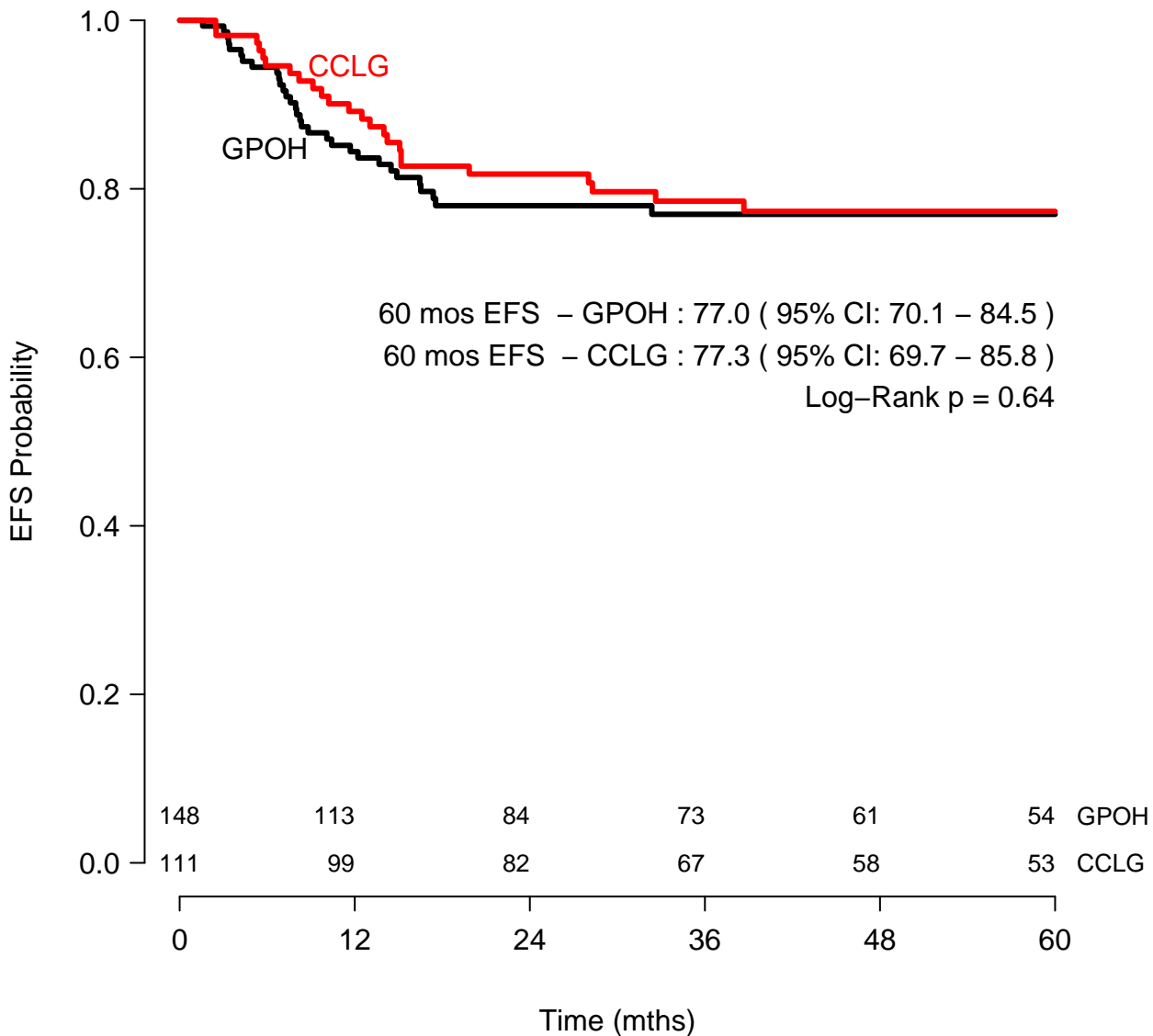

Supplement: Web figure 1e [file archdischild-2015-309212-s6.pdf]

## metastatic disease

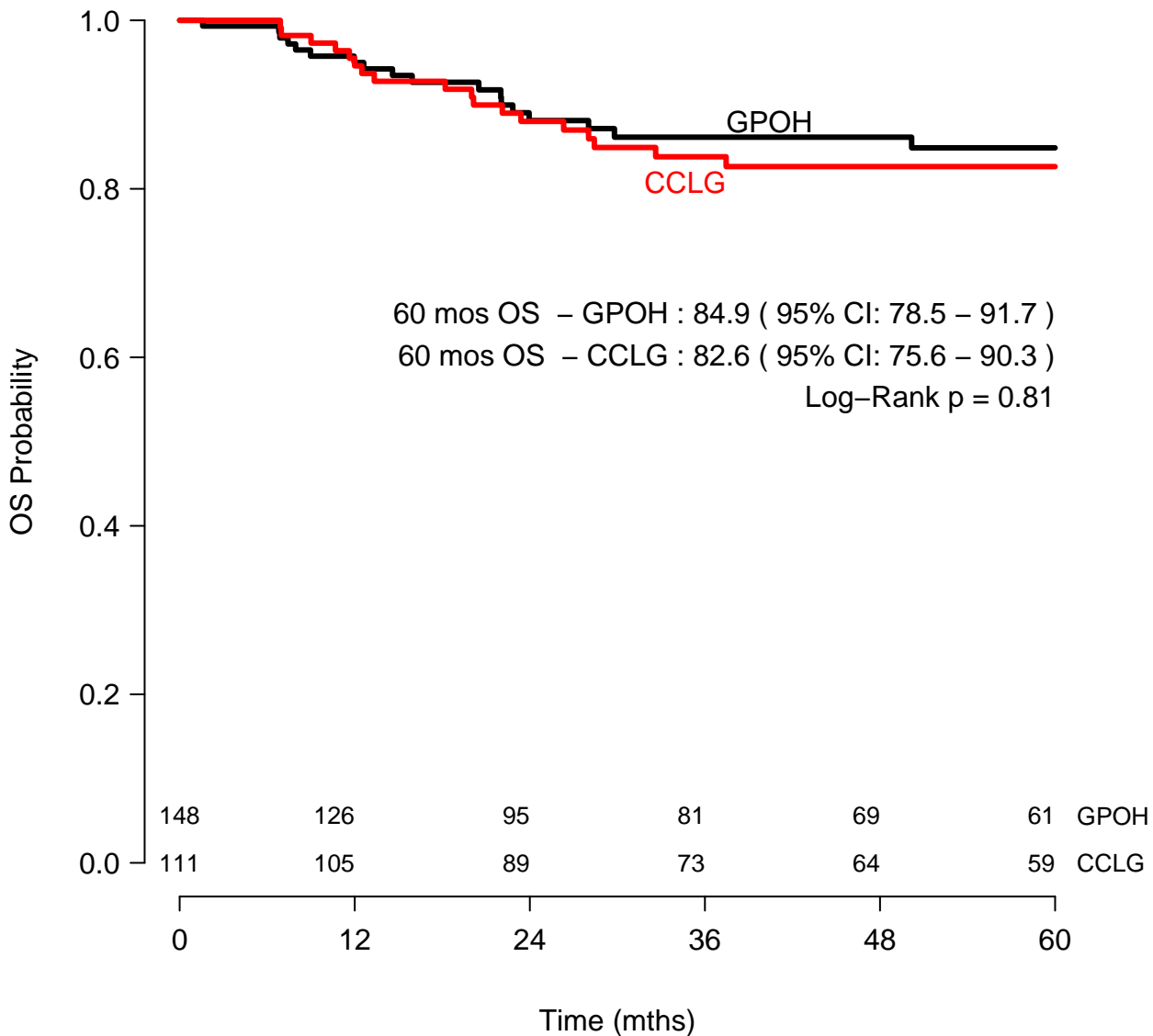

Supplement: Web figure 1f [file archdischild-2015-309212-s7.pdf]

Supplementary Figure 2:

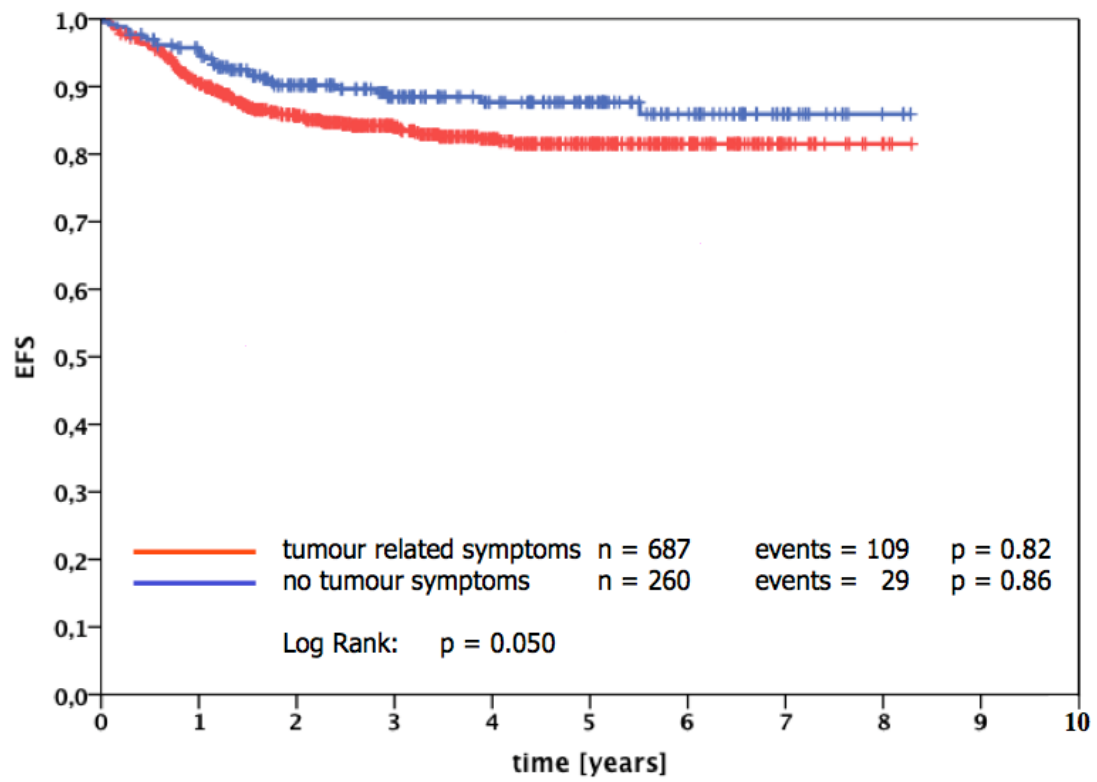

Supplement: Web figure 2 [file archdischild-2015-309212-s8.pdf]
